# Draft genome sequence of a methicillin-resistant *Staphylococcus haemolyticus* isolated from a circulating banknote in Bangladesh

**DOI:** 10.1128/mra.00355-26

**Published:** 2026-07-24

**Authors:** Arnob Saha, Mahir Anjum, Rafsun Jany Rahat, Md. Mehedi Hasan, Shyrin Jebun Labiba, Mst. Minara Khatun, Md. Ariful Islam

**Affiliations:** 1 Department of Microbiology and Hygiene, Faculty of Veterinary Science, Bangladesh Agricultural University, Mymensingh, Bangladesh; 2 Faculty of Veterinary Science, Bangladesh Agricultural University 54492, Mymensingh, Bangladesh; 3 Department of Pharmacology, Faculty of Veterinary Science, Bangladesh Agricultural University 54492, Mymensingh, Bangladesh; 4 Department of Genetic Engineering and Biotechnology, University of Chittagong 54493, Chittagong, Bangladesh; Portland State University, Portland, Oregon, USA

**Keywords:** *Staphylococcus haemolyticus*, whole-genome sequencing, methicillin resistance (MRSH), circulating banknote, Bangladesh

## Abstract

We report the draft genome of *Staphylococcus haemolyticus* AIL_BAU_sh26 from a circulating banknote in Bangladesh. The 2.48 Mb genome (32.6% GC; 79 contigs; N50 89,821 bp) encodes 2,487 coding sequences, 34 tRNAs, and 3 rRNAs. Multiple antimicrobial resistance determinants, including *mecA*, and a plasmid replicon were identified, underscoring the public health relevance of environmental staphylococcal genomic surveillance.

## ANNOUNCEMENT

*Staphylococcus haemolyticus* is an opportunistic, coagulase-negative staphylococcus that is linked to bacteremia, endocarditis, and skin and soft tissue infections, as well as device-related infections, especially in hospitalized or immunocompromised patients (1, 2). Since coagulase-negative staphylococci are also capable of acting as a reservoir of antimicrobial resistance determinants (3), their presence in circulating banknotes is of public health concern (4–6). Here we report the draft genome sequence of a methicillin-resistant *S. haemolyticus* strain isolated from a banknote in Bangladesh, contributing to the growing genomic data set of environmental antimicrobial resistance.

On 4 January 2026, strain AIL_BAU_sh26 was obtained from a circulating 500-taka banknote taken in one of the medicine shops of Charpara, Mymensingh, Bangladesh. The banknote surface was swabbed using a sterile cotton swab pre-moistened with sterile 0.85% NaCl. The swab was transferred to tryptic soy broth (Sigma-Aldrich, USA) and enriched aerobically at 37°C for 18 h prior to plating. Primary isolation was performed on 5% sheep blood agar (Oxoid, UK), and the culture was subcultured on mannitol salt agar (HiMedia, India) to selectively differentiate staphylococci. The culture was incubated aerobically at 37°C for 24 h. Culture characteristics, staining morphology, catalase, and PCR were used to identify a presumptive staphylococcal isolate. All bioinformatic tools were run with default parameters unless otherwise specified. Species and methicillin-resistance identification was performed by multiplex PCR targeting *mvaA* and *mecA* (7), and by a separate PCR targeting sodA (8), following the exact thermocycling conditions described in those publications.

DNA was extracted from a pure culture of strain AIL_BAU_sh26 using the DNeasy Blood and Tissue Kit (QIAGEN, Germany) according to the manufacturer’s instructions. Paired-end sequencing libraries were prepared using the Illumina DNA Prep Kit (formerly Nextera DNA Flex) following the manufacturer’s standard protocol. Whole-genome sequencing was done using the Illumina NextSeq 500 platform with 300-cycle chemistry. Paired-end sequencing (2 × 150 bp) generated 882,885 read pairs. Raw reads were quality checked with FastQC v0.12.1 and trimmed with fastp v0.23.4 before *de novo* assembly with SPAdes v3.15.2 (9). Assembly quality was assessed with QUAST v5.2.0 (10). Taxonomic classification with Kraken2 v2.1.3 (11) was confirmed by FastANI v1.34 (12), which showed 99.39% average nucleotide identity and 95.59% alignment coverage with *Staphylococcus haemolyticus*. The draft genome comprised 2,482,851 bp in 79 contigs, with 32.6% GC content, 56.25× coverage, and an N50 of 89,821 bp. The draft assembly was annotated in-house using Prokka v1.14.6 (13). The deposited GenBank record was subsequently re-annotated by NCBI using PGAP, consistent with standard deposition practice. Genome completeness was assessed with CheckM v1.2.2, yielding 98.7% completeness and 0.4% contamination. AMRFinderPlus v4.2.7 (14) detected *mecA*, *blaZ*, *blaI*, *blaR1*, *mph(C)*, and *msr(A)*, whereas no high-confidence hypervirulence markers were identified. PlasmidFinder v3.0.1 (15) detected the rep5e (SAP018B-type) plasmid replicon. The detection of mecA, together with macrolide and beta-lactamase resistance determinants, in an environmental *S. haemolyticus* strain recovered from a banknote highlights the role of currency as a potential vehicle for the dissemination of clinically relevant antimicrobial resistance genes in community settings. The assembly and annotation features are summarized in Table 1.

**TABLE 1:** Assembly and annotation features of *Staphylococcus haemolyticus* AIL_BAU_sh26

Features	Value
Sequencing platform	Illumina NextSeq 500 (300-cycle chemistry)
Library preparation kit	Illumina DNA Prep Kit (formerly Nextera DNA Flex)
Reported mean genome coverage	56.25×
Draft genome size (bp)	2,482,851
No. contigs (>500 bp)	79
GC content (%)	32.6
N50 (bp)	89,821
L50	9
Largest contig (bp)	294,369
Ambiguous bases (total N)	400
Genome completeness (%) (CheckM v1.2.2)	98.7
Contamination (%) (CheckM v1.2.2)	0.4
Protein-coding sequences	2,487
tRNA genes	34
rRNA genes	3
tmRNA genes	1
Hypothetical proteins	851 (35.2%)
AMR genes detected	*mecA*, *blaZ*, *blaI*, *blaR1*, *mph(C*), *msr(A*)
Plasmid replicon detected	rep5e (SAP018B type)
Hypervirulence markers	None detected

## Data Availability

The whole-genome shotgun project for *Staphylococcus haemolyticus* AIL_BAU_sh26 has been deposited at DDBJ/ENA/GenBank under accession number JBVYHG000000000. The version described in this paper is JBVYHG010000000. The project is available under BioProject PRJNA1426720 and BioSample SAMN55879617. Raw sequencing reads are available in the Sequence Read Archive under accession number SRR37316912.
